# Isolation of an Aptamer that Binds Specifically to *E*. *coli*

**DOI:** 10.1371/journal.pone.0153637

**Published:** 2016-04-22

**Authors:** Soledad Marton, Fernanda Cleto, Marco Aurélio Krieger, Josiane Cardoso

**Affiliations:** 1 Instituto de Biologia Molecular do Paraná, Department of Research and Development, 3375 Professor Algacyr Munhoz Mader Street, Curitiba, Brazil; 2 Instituto Carlos Chagas, Laboratório de Genomica Functional, 3375 Professor Algacyr Munhoz Mader Street, Curitiba, Brazil; University of São Paulo, BRAZIL

## Abstract

*Escherichia coli* is a bacterial species found ubiquitously in the intestinal flora of animals, although pathogenic variants cause major public health problems. Aptamers are short oligonucleotides that bind to targets with high affinity and specificity, and have great potential for use in diagnostics and therapy. We used cell-based *S*ystematic *E*volution of *L*igands by *EX*ponential enrichment (cell-SELEX) to isolate four single stranded DNA (ssDNA) aptamers that bind strongly to *E*. *coli* cells (ATCC generic strain 25922), with Kd values in the nanomolar range. Fluorescently labeled aptamers label the surface of *E*. *coli* cells, as viewed by fluorescent microscopy. Specificity tests with twelve different bacterial species showed that one of the aptamers–called P12-31—is highly specific for *E*. *coli*. Importantly, this aptamer binds to Meningitis/sepsis associated *E*. *coli* (MNEC) clinical isolates, and is the first aptamer described with potential for use in the diagnosis of MNEC-borne pathologies.

## Introduction

*Escherichia coli* is a Gram-negative bacterial species found ubiquitously in the intestinal gut flora of animals, including humans, and can also survive and multiply in abiotic environments. Typically, it colonizes the infant gastro-intestinal tract within hours after birth, with lifelong benefits to the host [1]. However, *E*. *coli* can acquire specific virulence factors that allow it to adapt to new niches, causing diseases that are major public health issues, with high morbidity and mortality [1]. Pathogenic *E*. *coli* variants can cause three clinical syndromes: 1) sepsis/meningitis (meningitis/sepsis associated *E*. *coli*, or MNEC), 2) urinary tract infections (uropatogenic *E*. *coli*, or UPEC), 3) and intestinal and diarrheal diseases. Depending on the disease type, pathogenic *E*. *coli* in the intestinal tract can be further be classified in at least six different “pathotypes”: enterohemorragic (EHEC), enteropathogenic (EPEC), enterotoxigenic (ETEC), enteroaggregative (EAEC), diffusely-adherent (DAEC) and enteroinvasive *E*. *coli* (EIEC) [2].

Currently used *E*. *coli* detection methods normally require an enrichment step of bacterial culturing, which usually takes 2 to 3 days to yield results. This method does not permit rapid bacterial detection, which requires bacterial identification either by the polymerase chain reaction (PCR) [3, 4], or by the specific recognition of bacterial cell surface biomarkers using biological probes, including antibodies and aptamers [5], which enable bacterial detection in few hours.

Aptamers are short oligonucleotides that bind targets with high specificity and affinity (in the nanomolar or picomolar ranges) [6], and are isolated by *S*ystematic *E*volution of *L*igands by *EX*ponential enrichment (SELEX) procedures [6–8]. SELEX methodologies follow the same pattern: a large pool of random sequences is subjected to iterative steps of selection and amplification, obtaining a pool of molecules enriched for those with high affinity and specificity to the target. At the end of the process, sequences are cloned and evaluated individually for their binding properties [6–8].

Aptamers have been selected for targets with varying degrees of complexity, from small molecules to whole cells or tissues [9]. Cell SELEX methods have the advantage of selecting multiple aptamers that could potentially bind to different targets on the cells in their native conformation and physiological environment, without the requirement for target protein purification before selection. Cell SELEX also allows aptamer selection without previous knowledge of the cellular target, and eliminates the possibility of selecting aptamers that bind to targets that are not exposed on the native cell surface [10, 11].

Aptamers have high potential as diagnostic and therapeutic tools, with many advantages when compared with antibodies [12], including their smaller size–which improves access to biological environments ‘hidden’ from antibodies–their lack of immunogenicity, and the lower cost and higher reproducibility of nucleotide production. In addition, aptamers can be chemically modified to became more stable [13], labeled with fluorophores or other reporters [14], and can be easily truncated to eliminate sequences not important for interaction [15, 16]. These valuable properties make aptamers flexible and powerful tools for diagnostic and therapeutic purposes [5, 9, 12, 16–20].

Single strand DNA (ssDNA) aptamers have been selected against cells from different bacterial species, including *Escherichia coli* K88 [21] *and* NSM59 [22], a fecal *E*. *coli* isolate [23], *Salmonella typhimurium* [24–26], *S*. *enteritidis* [27, 28], *S*. *paratyphi* A [29], *Salmonella* O8 [30], *Vibrio parahaemolyticus [31, 32], Listeria monocytogenes [33, 34], Shigella dysenteriae [35], Streptococcus mutans [36]*, *Streptococcus pyogenes [37], Staphylococcus aureus [38], Proteus mirabilis [39], Pseudomonas aeruginosa [40], Mycobacterium tuberculosis [41], Francisella tularensis* subspecies (subsp.) japonica [42] and *Campylobacter jejuni [43].* Besides cell SELEX, surface molecules have been used for ssDNA aptamer selection, including peptidoglycan expressed in all bacterial cells [44], lipopolysaccharides found in Gram-negative cells [45], or specific surface proteins such as K88 frimbiae from *E coli* [46] or outer membrane proteins [47]. Aptamers that recognize bacterial cells have been used in biosensors devices allowing specific and rapid detection of bacterial cells [48]. Biosensors are composed of a biomolecule (typically an aptamer or antibody) that recognizes the target analyte and a transducer that converts the recognition event into a measurable signal. Thus far, biosensors using aptamers have been described that against *S. enteriditis [49–51]*, *S*. *typhimurium* [52–56], *Vibrio parahaemolyticus [52], Staphylococcus aureus [53, 57–59]* and *E. Coli [51, 60, 61]* have been designed an binding have been measure by changes in electrical/electrochemical, optical, and mass sensitive parameter when the bacteria is bound. However, no biosensors described to date bind to MNEC species of *E*. *coli*.

In the present work, we describe the selection of ssDNA aptamers against the generic non-virulent strain *E*. *coli* ATCC 25922, using cell SELEX. We isolated four aptamers that bind to *E*. *coli* with high affinity, with Kd values in the nanomolar range. All four aptamers bind to the surface of *E*. *coli*, and one of the aptamers shows high specificity for *E*. *coli* cells. Importantly, this aptamer also binds to three MNEC clinical isolates from septic patients. To our knowledge, this is the first description of an aptamer capable of recognizing MNEC.

## Materials and Methods

### Bacterial strains and culture

The bacterial strain *E*. *coli* ATCC 25922 (American Type Culture Collection, Georgetown, DC, USA) was maintained in Luria-Bertani (LB) medium. Whole bacterial cells to be use as targets for selection were cultured at 37°C to a OD_600_ of 0.3 (equivalent to ~2.4 x 10^8^ bacteria/mL), washed twice with PBS (NaCl_2_ 137 mM, 2.7 mM KCl, 4.3 mM Na_2_HPO_4_.7H_2_O, 1.5 mM KH_2_PO_4_) and diluted in selection buffer (PBS containing 1.4 mM MgCl_2_).

The following bacterial strains were used in binding assays: *Klebsiella pneumonia* ATCC 27853, *Enterobacter aerogenes* ATCC 13048, *Proteus mirabilis* ATCC 00557, *Pseudomonas aeruginosa* ATCC 700603, *Staphylococcus aureus* ATCC 29213 and *Enterococcus faecalis ATCC* 29212, from ATCC; and *Enterobacter cloacae*, *Proteus vulgaris*, *Morganella morganii*, *Citrobacter freundii*, *Acinetobacter baumannii*, *Enterococcus faecalis*, *Enterococcus faecium*, from the bacterial collection of the Instituto Adolfo Lutz (São Paulo, SP, Brazil). The MNEC clinical isolates used here were obtained from the Clinical Hospital of the Federal University of Paraná (HC, Curitiba, PR, Brazil).

### Random library, primers and aptamers

The single stranded DNA (ssDNA) library used here represents a collection of 88-mer nucleotides with a central random region of 40 nt (N_40_) flanked by two 24-nt primer-binding sites (5’ CATACGATTTAGGTGACACTATAG-**N**_**40**_-ATTTCTCCTACTGGGATAGGTGGA 3’) obtained from Integrated DNA Technologies (Coralville, IA, USA). The BR Forward (5’CATACGATTTAGGTGACACTATAG3’) and reverse (5’TCCACCTATCCCAGTAGGAGAAAT3’) primers, selected aptamers and the random oligonucleotide 5’CATACGATTTAGGTGACACTATAGTTCCAACATAGTGTCTGATTTTCTTAATGGTAGGCGAGTAATTTCTCCTACTGGGATAGGTGGA3’ (either unlabeled or 5’-labeled with 6-FAM) were synthesized by Integrated DNA Technologies. The 183-mer ssDNA AT1 (5’CCACTCATGTGAGAGCCAATTGTGAAGAGCACAAAAGGTGATTTCATTTCCTTTTGTGTAATTTGCATGTTTGAACAGACACTGTATCTGTATTGTTACAATGGATATTGATTTGGTGTTTGCAGGATCCTGGACAGAAGCAAAGGCAAAGGTATAAAAGATTTGATCCCATTAGTGTCCAAC3’) was used as an exogenous control, and was amplified using the AT1 forward (5’CTCATGTGAGAGCCAATTGTGAAG3’) and AT1 reverse (5’GGACACTAATGGGATCAAATCTTTTATACC3’) primers.

### Cell-SELEX

**Fig 1:**
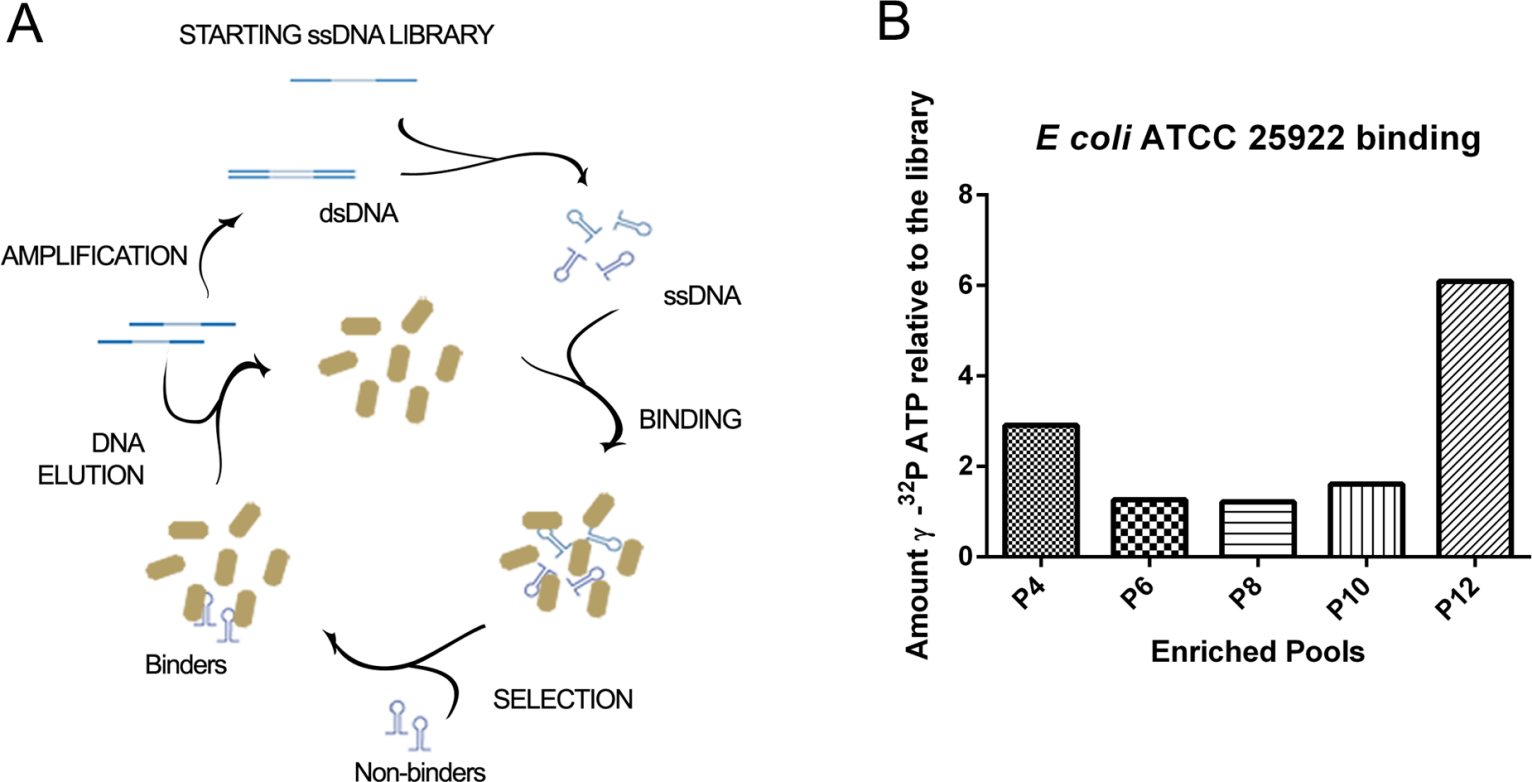
Enrichment of *E*. *coli*-binding aptamers by cell SELEX. (A) Schematic representation of the *in vitro* selection of aptamers against *E coli* ATCC 25922. *E coli* bacterial cells were incubated with an ssDNA random library for 45 min at room temperature. Then, the bacterial suspension was centrifuged, washed repeatedly, and the molecules that remained bound to bacterial cells were eluted at 95°C. The recovered aptamers were amplified by asymmetric PCR, and then incubated with bacterial cells in the next round of selection. (B) Enrichment of *E coli* binding ssDNA pools during 12 iterative rounds of aptamer selection. Bacterial cells were incubated with γ ^32^P ATP-labeled ssDNA from rounds 4 (P4), 6 (P6), 8 (P8), 10 (P10) and 12 (P12) of selection, and then bound aptamers were eluted and quantified by scintillation counting. The graph represents the fold increase of bound radiolabeled ssDNA from each round relative to that of the starting library (P0).

A scheme of the bacterial cell-SELEX process is shown in Fig 1A. Briefly, 1.5 nmol of random library nucleotides (approx 10^14^ molecules) diluted in selection buffer were denatured for 5 min at 95°C, cooled for 15 minutes at 25°C and incubated with 1 mL of 10^7^ colony forming units (CFU)/mL of *E*. *coli* ATCC 25922 cells for 45 min, at room temperature, and under constant agitation (220 rpm). Then, the bacterial suspension was centrifuged for 6 minutes at 8,000 g. The supernatant was discarded, unbound ssDNA sequences were removed by washing with 1 mL of selection buffer, and bacterial cells were centrifuged for 6 min at 8,000 g. Bound ssDNA was eluted by resuspending the bacteria-aptamer complex in sterile water and heating at 95°C for 5 min. The supernatant was collected and amplified by asymmetric PCR in a 100 ul reaction, using GoTaq® Green Master Mix (Promega, Fitchburg, WI, USA), with 0.06 μM reverse BR primer and 2 μM forward BR primer. PCR was performed by forty cycles of 1 min of denaturation at 95°C, 30 seconds of annealing at 65°C, and 40 seconds of extension at 72°C. The ssDNA was extracted from 8% polyacrylamide native gels colored with SYBR® Gold Nucleic Acid Gel Stain (S1 Fig, Invitrogen, Carlsbad, CA, USA), precipitated with ethanol and resuspended in selection buffer to be used in the next selection round. A total of twelve rounds were performed and the selection conditions were changed during the cell-SELEX procedure in order to increase the selective pressure towards the last steps (S1 Table).

PCR products obtained after twelve selection rounds were cloned using the TOPO® TA Cloning® Kit (Invitrogen), and positive clones were sequenced. Aptamers sequences were analyzed using the CLustal and Mfold structure prediction algorithms.

### Pool binding assays

The ssDNA random library and enriched nucleotide pools from each round of selection were amplified by asymmetric PCR, purified from polyacrylamide native gels and radiolabeled with γ ^32^P ATP. The 5´end-labeling reactions were performed in a 15-μL reaction mix containing the T4 polynucleotide kinase (Invitrogen), following the manufacturer’s instructions. Samples were incubated for 1 hr at 37°C, and then the enzyme was inactivated by heating for 5 min at 80°C. Unincorporated γ ^32^P ATP was eliminated by DNA purification through sephadex G-25 columns (GE Healthcare, Little Chalfont, Buckinghamshire, UK).

To determine the binding capability of the pools 10^6^ CFU/mL of *E*. *coli* cells were incubated with 50 nM of enriched pools or ssDNA random labeled library and agitated at room temperature for 45 min, in the presence of the selection buffer with 0.05 μg/μl of BSA. Following three washes with 1 mL of selection buffer, by centrifugation at 8,000 g, 6 min, the amount of radiolabeled ssDNA associated with bacterial cells was measured by scintillation counting. The amount of radiolabeled ssDNA bound to bacterial cells at each round of selection was normalized to the radiolabeled library signal.

### Aptamer binding analysis by qPCR

For the identification of individual aptamers, 10^6^ bacterial cells were incubated with 200 nM of aptamer or random library in the presence of selection buffer with 0.05% BSA, for 45 min at room temperature, and under agitation (at 220 rpm). The bacterial aptamer complexes were washed three times with 1 mL of selection buffer and resuspended in 25 μl of sterile water. Before the elution step, 0.016 pmol of the AT1 oligonucleotide, used as exogenous control, was added to each sample, and aptamers were eluted by heating for 5 min at 95°C, followed by centrifugation for 5 min at 8,000 g. ssDNA aptamers recovered in the supernatant were quantified by SYBR Green-based qPCR, using an Applied Biosystems 7500 Real-Time PCR system (Applied Biosystems, Foster City, CA, USA). All reactions were done in 20 μl volume, and in triplicates in 96-well plates, using BR forward and reverse primers, for aptamer or random library amplification, or AT1 forward and reverse primers, for AT1 amplification.

Aptamer Kd determination was performed by qPCR of the aptamer bound to bacterial cells after the incubation of 10^6^ bacterial cells with increasing concentration of the aptamer (6.25 nM, 12.5 nM, 25 nM, 50 nM, 100 nM, 200 nM and 400 nM), followed by washing steps. Apparent Kd values for each aptamer were determined by Linewaver-Burk analysis, using the formula:

1/[complex] = Kd/ [Cmax] x 1/[aptamer] + 1/[Cmax], where kd is the steady state dissociation constant, [Complex] is the concentration of the complex bacteria-aptamer, [Cmax] is the concentration of the complex at maximal binding capacity, where all the binding sites are occupied by the aptamer, and [aptamer] is the concentration of the aptamer.

The estimated number of binding sites/*E Coli* calculation was made according to [62].

### Fluorescence assays

Bacterial cells were washed once and resuspended in PBS at a density of 10^9^ CFU/ml, and then incubated for 45 min with 400 nM of 6-FAM-labeled aptamers (5’ labeling). Then, bacteria-aptamer complexes were adhered to poly-L-lysine-coated slides for 20 min (at room temperature), slides were washed 3 times in PBS and observed in a Nikon Eclipse E600 epifluorescence microscope, using a 100x objective.

### Secondary structure prediction

To predict aptamer secondary structure, we used the mfold algorithm [63], set for the conditions used in binding assays (1.4 mM MgCl_2_ and 137 mM NaCl, at 25°C).

## Results

### Sequential steps of cell-SELEX yields aptamer pools with increased binding to *E*. *coli*

To select aptamers that bind to *E*. *coli* we performed a cell-SELEX strategy as previously described [38, 40, 64, 65] (Fig 1A). We chose to use in our experiments the *E*. *coli* strain ATCC 25922 (serotype O6 and biotype 1), because this strain—originally isolated from a human clinical sample (Seattle, WA, USA; 1946)—is commonly used for quality control of antibody sensitivity assays. Also, we used live, unfixed bacterial cells, since formaldheide, glutaraldeide or alcohol fixation may alter the conformation of cell wall molecules [66], the most likely aptamer targets. The composition of the bacterial cell wall changes during growth in culture [38, 67]. Thus, to minimize differences in cell surface composition, we used exponential-phase bacterial cells at in all the experiments. Also, we used non-modified nucleotides due to the lower costs and ease of production with conventional enzymes, and preferred ssDNA to RNA, due the higher stability of DNA molecules.

After each of the twelve rounds of positive selection, the enrichment of ssDNA molecules that bind bacterial cells specifically was monitored using γ ^32^P ATP 5’-end labeled oligonucleotide pools [68]. Radiolabeled ssDNA that binds to bacterial cells was quantified by scintillation counting, and the results were reported relative to the binding of the radiolabeled starting library binding (Fig 1B). The results show that the pool of oligonucleotides recovered from the twelfth round of selection (‘P12’) had approximately 6-fold higher binding to *E coli* cells than the starting library pool. These results indicate that P12 was the most enriched in *E coli*-binding ssDNA molecules.

The pool of molecules from the fourth round of selection (P4) displayed about 3-fold increase in binding to *E coli* relative to the starting library. Then this binding increase was lost in P6, P8 and P10 indicating that these pools were not enriched in *E coli* binding molecules than the library pool.

### Round 12 of cell SELEX contains *E*. *coli*-binding aptamers

To identify aptamer sequences that specifically recognize *E*. *coli* ATCC 25922, we cloned and sequenced the aptamers found in pool P12 (Fig 1 and Table 1). We obtained 64 clones, and primary sequence similarity analysis identified eleven highly related families of aptamers within the group (Table 1), corresponding to the 34% (22 aptamers) of all cloned sequences, while the remaining 66% of sequences were not clearly related to each other.

**Table 1:** Sequence families in P12 aptamer pool.

Aptamer clone	Central sequence of families of aptamers
P12-3 P12-34	GCGGATGCGTGAGACCCCCACAAGCAGTGAGTAGGAGGGGACGGATGCGTGAGACCCCCACAAGCAGTGAGTAGGAGGGG
P12-9 a, P12-9 b	TGCGGGCGGAGGACACGGACCCGTATGGGAGCAATGCACG
P12-11 P12-31	CCCTCCGGGGGGGGGGGTCATCGGGATACCTGGTAAGGATACCCTCCGGGGGGG———TCATCGGGATACCTGGTAAGGATA
P12-15 a, P12-15 b	GGCCACCCACAGGCACTCCGCTCATGAATCGTGGAGTCGG
12–17 a, P12-17 b	GACGGTGGCAGGGAAAGGGGTCGGGCATATGGCGGAGGGG
P12-21 P12-55	CCGGAGGTGGGTGAGGTCTGCGGCAGGCTGTGTGGGTGGACCGGAGGGGGGTGAGGTCTGCGGCAGGCTGTGTGGGTGGA
P12-30 a, P12-30 b	CACCACGGACACGATCCCAAGCTAGGAGGTGCGGCGGGGT
P12-48 a, P12-48 b	TGGCACAGCACGTCGCACGGTCCCCGGGAGGTGTTCACTG
P12-51 a, P12-51 b	TCGCGAGTGCGTGTACGCCACACATCACAAAAGGGGTGTG
P12-52 a, P12-52 b	CCGCCCAGCGGGGGTAGGGCCGGACGTAGGAGGAGCTGCG
P12-66 a, P12-66 b	GGCAACATTCAGACATACCAGTACCCACTCGGACTTCCCG

a and b are clones who share the same sequence

**Fig 2:**
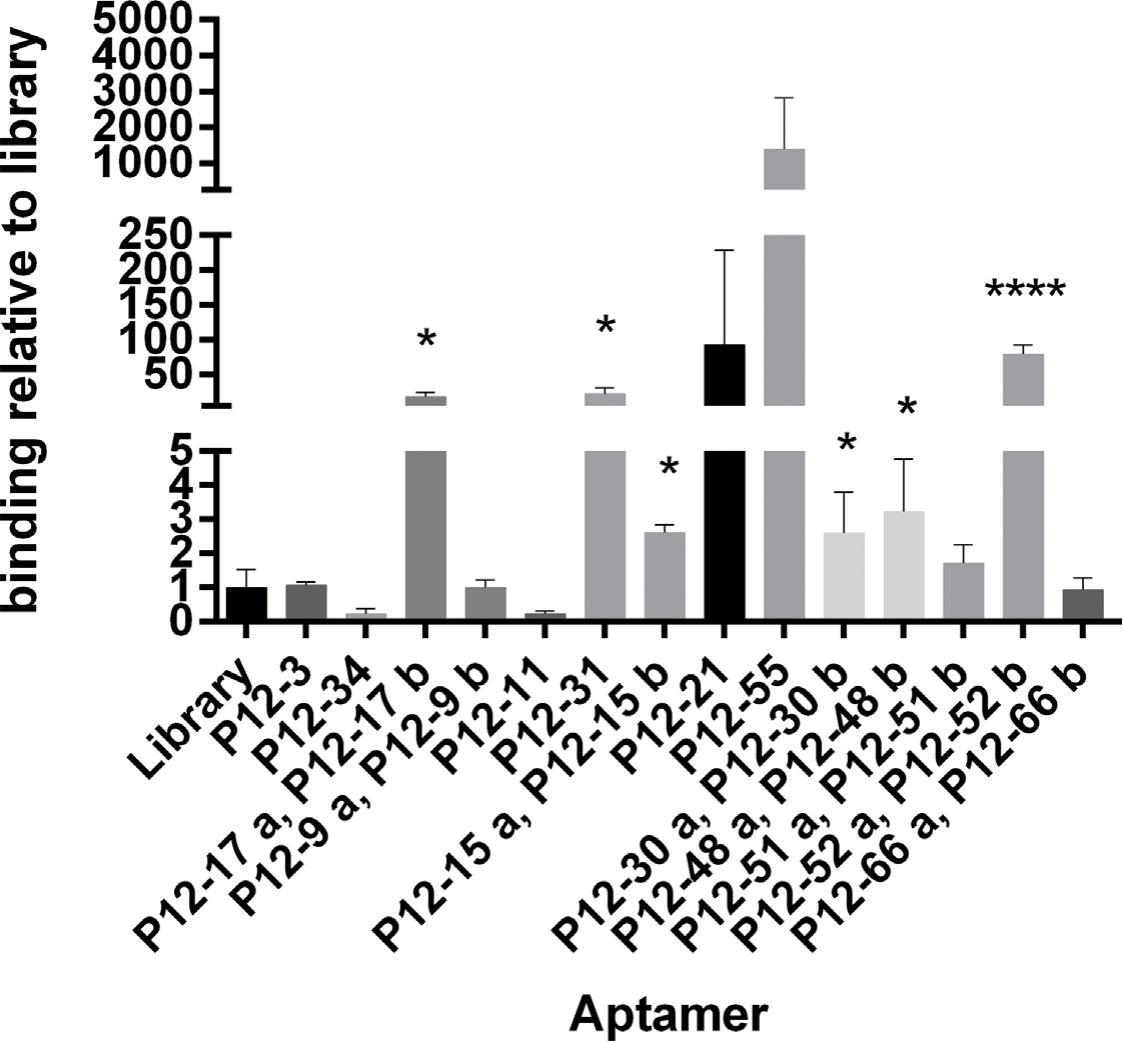
Binding to *E*. *coli* of the individual sequences from round 12 cell SELEX. Pool 12 (P12) aptamers with conserved sequences (within the pool) were incubated with *E coli* strain ATCC 25922 cells, and then bound aptamers were eluted and quantified by qPCR (using the ΔΔCt method). Results show the amount of bound aptamers relative to that of starting library nucleotides. Data represent mean ± SD of 3 independent experiments. *, p < 0.05 and ****, p<0.0001.

We tested the ability of related sequences to bind *E*. *coli*, compared with that of the random library nucleotides, by incubation with 10^6^ *E*. *coli* cells, followed by elution and ssDNA quantification by qPCR (Fig 2A). Five aptamers, - P12-17, P12-31, P12-52, P12-21 and P12-55 –strongly bind to *E*. *coli* ATCC 25922. In particular, the binding of P12-21, P12-51 and P12-55 to *E*. *coli* cells was more than 100-fold stronger than that of the random library, while that of P12-31 and P12-17 was 10-fold stronger than that of the random library. Other sequences showed low (P12-15, P12-30 and P12-48) or no detectable binding to *E*. *coli* bacterial cells (Fig 2A).

While the aptamers P12-17 and P12-52 were representatives of two groups of clones with identical sequences, the primary sequences of aptamers P12-31 and P12-55 are related to sequences P12-11 and P12-21, respectively (Table 1). P12-31 showed stronger binding than P12-11, and this difference could be explained by primary sequence changes. Relative to P12-11, P12-31 lacks four G nucleotides in positions 14 to 17 of the central region (Table 1). In contrast, the binding of P12-55 *E*. *coli*-binding was not significantly different to that of P12-21, in agreement with the near sequence identity between these oligonucleotides in the central region, with a single G-to-T substitution from P12-55 to P12-21 (Table 1).

**Fig 3:**
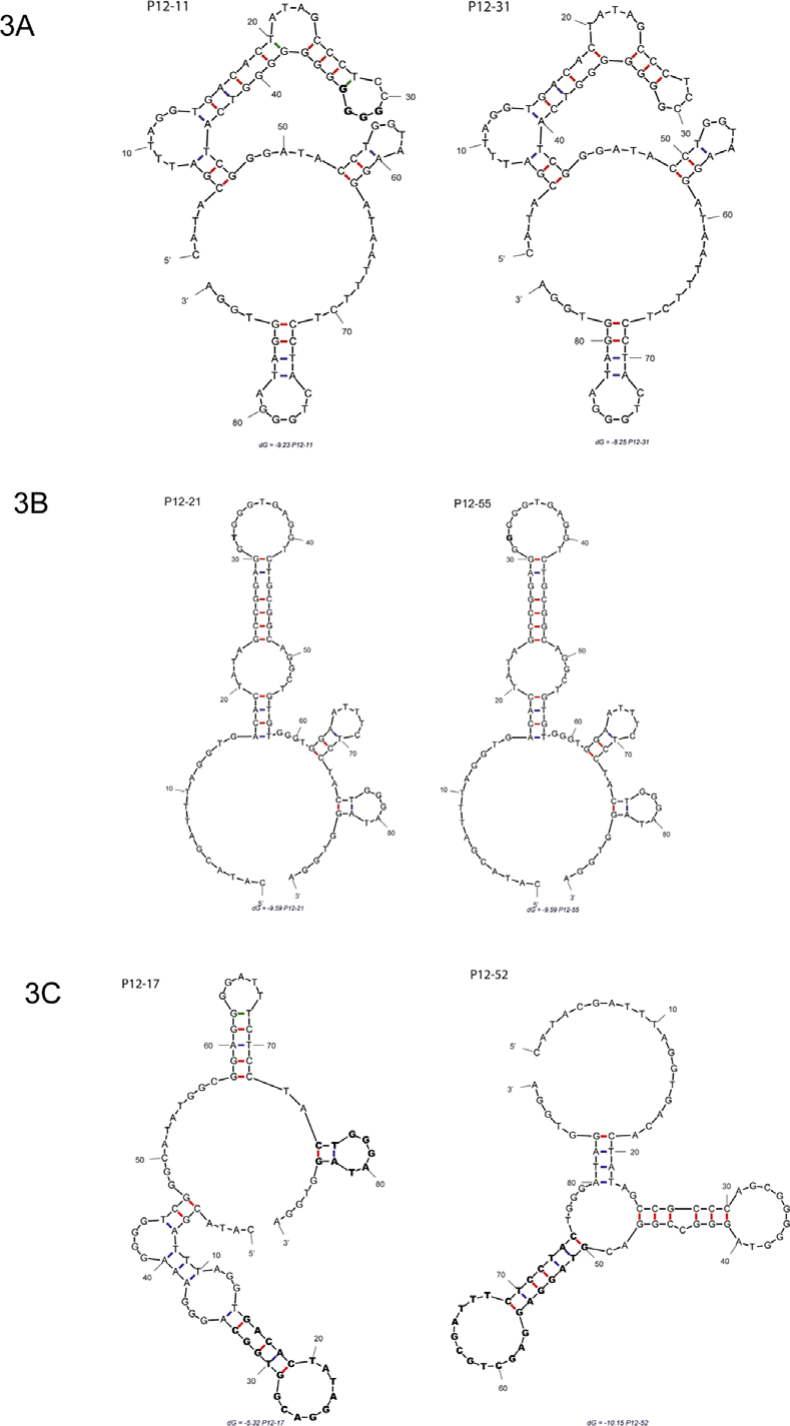
Prediction of pool 12 (P12) aptamer secondary structures. The structures of aptamers with the highest *E coli* binding values were predicted using the mfold algorithm [63]. (A) Predicted structures of similar aptamers P12-11 and P12-31. The four G residues in P12-11 that are missing in P12-31 are depicted in bold. (B) P12-21 and P12-55 predicted structures. The different nucleotide (G or T) in the apical loop is in bold. (C) Structures of one conformer of aptamers P12-17 and P12-52, with conserved stem loops were depicted in bold.

To understand the impact of aptamer sequences on binding, we generated predicted secondary structures for the most efficient *E*.*coli*-binding aptamers using the mfold algorithm [63]. P12-31 and P12-11 have similar predicted secondary structures, but the four G residues absent in P12-31 are predicted to alter the folding pattern of the main stem-loop region, changing the sequences of upper lateral and apical loops (Fig 3A). P12-55 and P12-21 have nearly identical predicted secondary structures, and the different nucleotide (G or T) is situated in the apical loop of the stem-loop structure (Fig 3B).

For the aptamers P12-55 and P12-31, mfold yielded only one predicted secondary structure, while different alternative conformers were predicted for P12-17 and P12-52 (S2A and S2B Fig). In fact, P12-17 has four different possible secondary structures; however, all of them have two stables stem loops (Fig 3C). The mfold results for P12-52 include at least five thermodynamically stable predicted structures that share one conserved stem loop (Fig 3B). The presence of stable stem loops in the predicted secondary structures of P12-17 and P12-52 suggests that this feature could play an important role in the interaction of aptamers with theirs ligands in the bacterial cell wall.

### Aptamers from the P12 pool have high affinity and are specific for *E*. *coli* ATCC 25922

**Fig 4:**
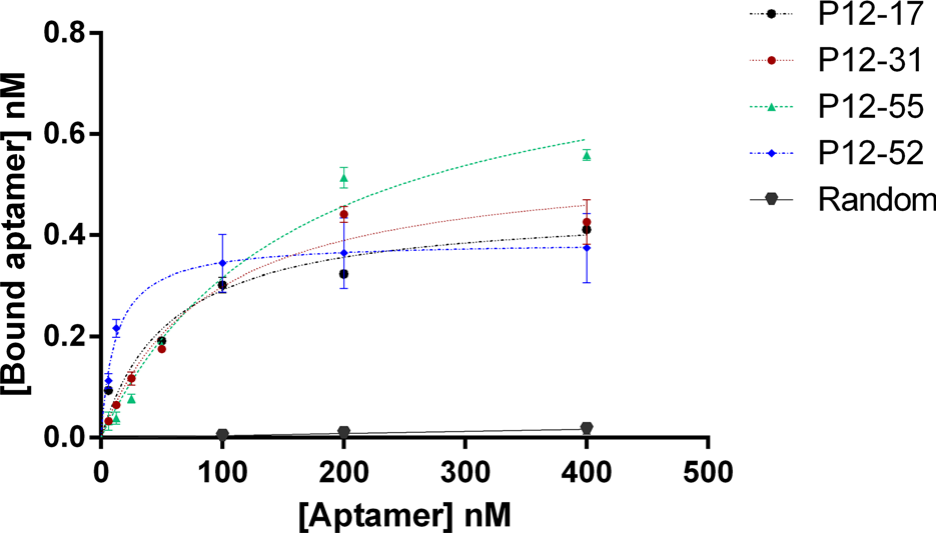
Binding affinity of selected pool 12 aptamers to *E coli* ATCC 25922. Binding affinity curves of aptamers P12-17, P12-31, P12-52 and P12-55 to live *E coli* ATCC 25922. Aptamer binding was quantified by qPCR. Data represent mean ± SD values of three independent experiments. The binding analysis was performed using GraphPad Prism 6, under the non-linear fit model for specific binding.

To evaluate aptamer binding in more detail, we determined the affinity for *E*. *coli* ATCC 25922 of the four P12 aptamers with the highest binding values relative to that of the original library. All of the four aptamers showed saturable binding to live bacterial cells, whereas a random oligonucleotide showed low or no detectable binding to *E*. *coli* (Fig 4). The dissociation constants (Kd) of the four aptamers were estimated to be between 11.97 (P12-52) and 161.0 nM (P12-31) (Table 2), and the Bmax values between 0.3874 (P12-52) to 0.8283 (P12-55) (Table 2). Based on the Bmax data, we calculated the estimated number of binding sites per bacterial cell [62]. Interestingly, P12-52 had the lowest estimated number of binding sites per *E*. *coli* cell *(*6996 ± 350), despite the fact that this aptamer had the highest affinity for these cells, with a low Kd value (11.97 nM).

**Table 2:** Binding characteristics of the selected aptamers.

Aptamer	Kd (nM)	Bmax (nM)	Estimated number of bindig sites/E coli
P12-17	56.33 ± 15.87	0.46 ± 0.03	8100
P12-31	87.03 ± 17.32	0.56 ± 0.04	10113
P12-52	11.97 ± 2.94	0.39 ± 0.02	6996
P12-55	161.0 ± 34.74	0.83 ± 0.07	14950

**Fig 5:**
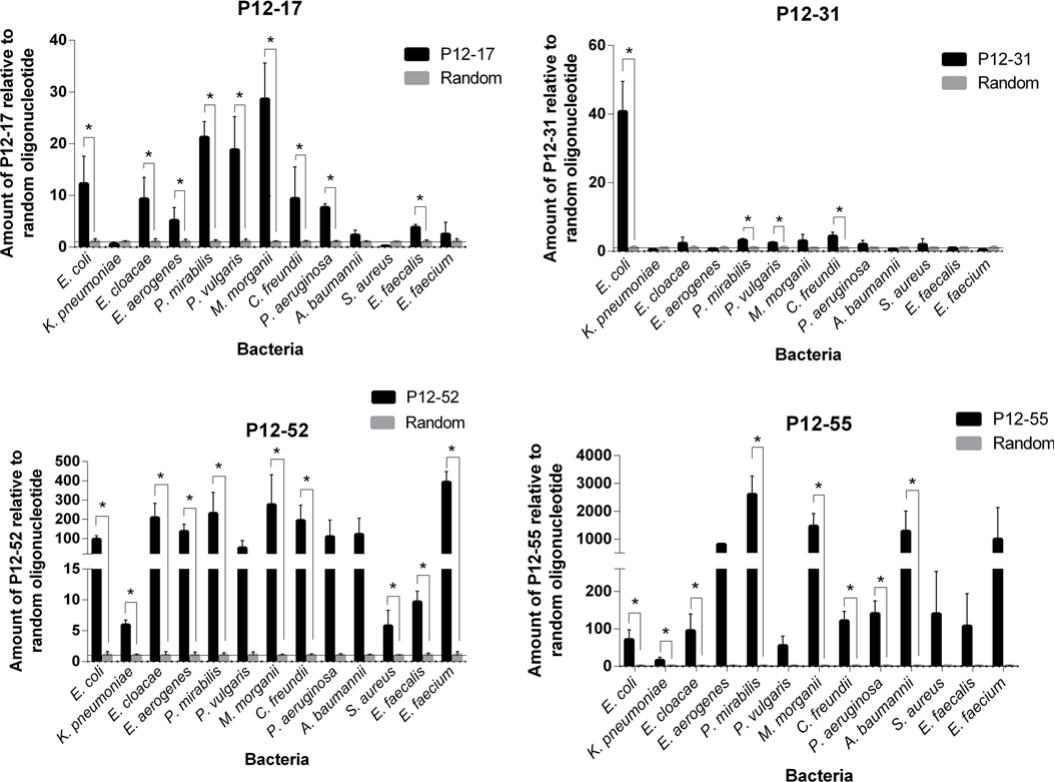
Specificity of the aptamers to *E*. *coli* ATCC 25922. Binding assays of P12-17, P12-31, P12-52 and P12-55 to the target species *E*. *coli* ATCC 25922 and to other (non-target) bacterial species. Bound aptamers were quantified by qPCR (ΔΔCt method) using a random oligonucleotide as a negative control, and AT1 as an exogenous reference control. Data represent mean ± SD of three independent experiments. * p<0.05.

We also evaluated the specificity of aptamers to *E*. *coli* by comparing the ability of these molecules to bind to 12 different bacterial species, including nine Gram-negative and three Gram-positive strains (Fig 5). The results showed that P12-17, P12-52 and P12-55 showed strong binding to bacterial species other than *E*. *coli*. Although P12-31 could bind to other Gram-negative bacteria (*P*. *mirabilis*, *P*. *vulgaris* and *C*. *freudini*), its binding to those species was weaker than that observed towards *E*. *coli *(using the Dunnett’s test, α = 0.05; S2 Table). P12-17 and P12-52 could recognize Gram-negative and positive bacterial cells. While P12-17 could only bind to *E*. *faecalis*, P12-52 bound strongly to all three Gram-positive bacteria tested (*S*. *aureus*, *E*. *faecalis* and *E*. *freudini*).

### Selected aptamers bind to the surface of live *E*. *coli* cells

**Fig 6:**
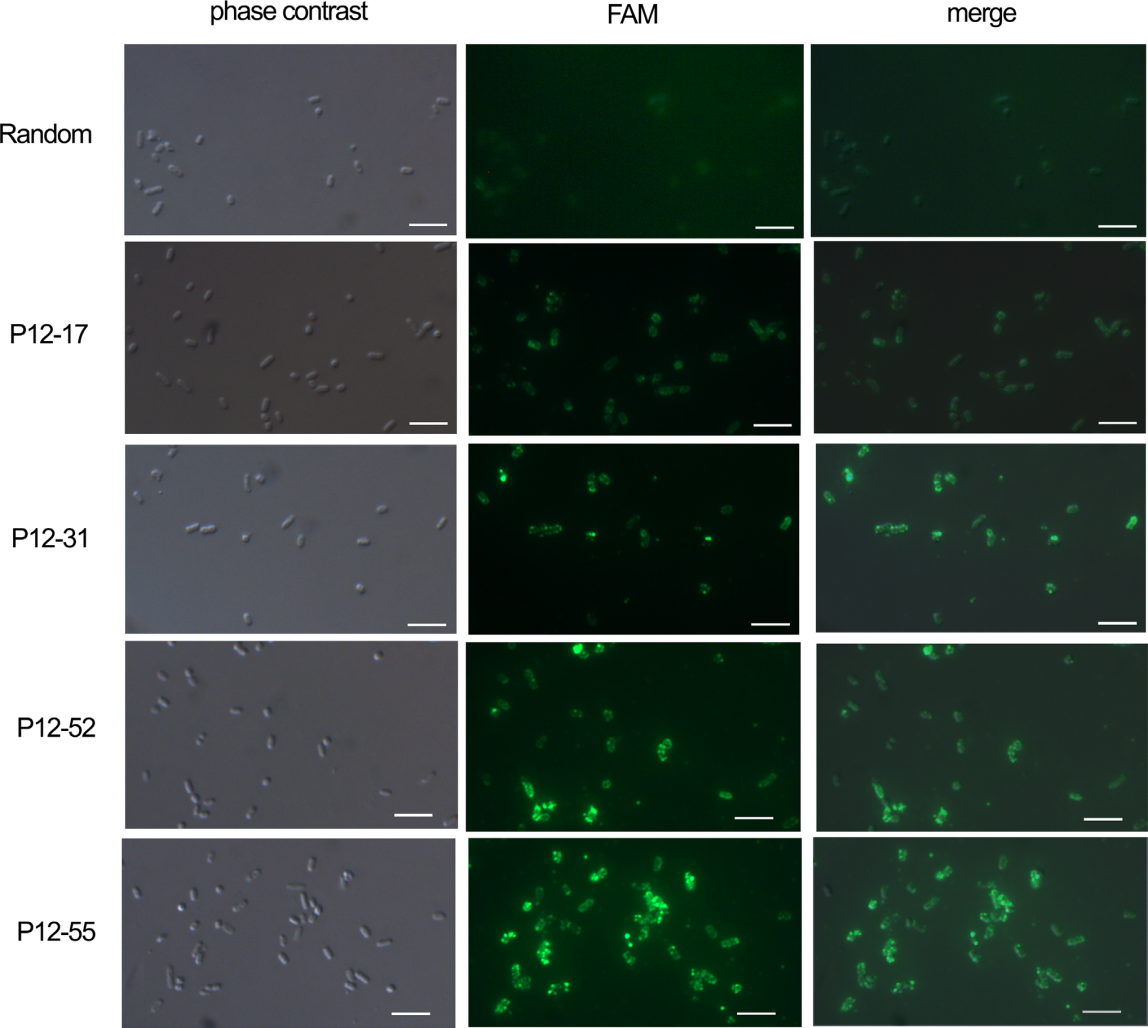
Fluorescence microscopic images of aptamer binding to *E coli* ATCC 25922. Bacterial cells were incubated with aptamers (400 nM) 5’-labeled with 6-FAM, and then fixed and adhered to poly-L-lysine-coated slides. A random oligonucleotide sequence was used as a nonspecific binding control. Scale bar, 4 μm.

To confirm that the selected aptamers were capable of binding *E*. *coli* cells, we visualized by fluorescence microscopy the binding of aptamers P12-17, P12-31, P12-52 and P12-55 to *E*. *coli* ATCC 25922 cells. Bacterial cells were incubated with 6-FAM labeled aptamers, adhered to poly-L-lysine-coated slides, and then visualized by fluorescence microscopy. We observed that the four aptamers bound to bacterial cells, whereas no clear labeling was detected using random ssDNA (Fig 6). For all aptamers, labeling appeared as granules covering bacterial cells (Fig 6), likely corresponding to aptamer binding to components of the bacterial surface.

### Aptamer recognition of *E*. *coli* isolates from septic patients

**Fig 7:**
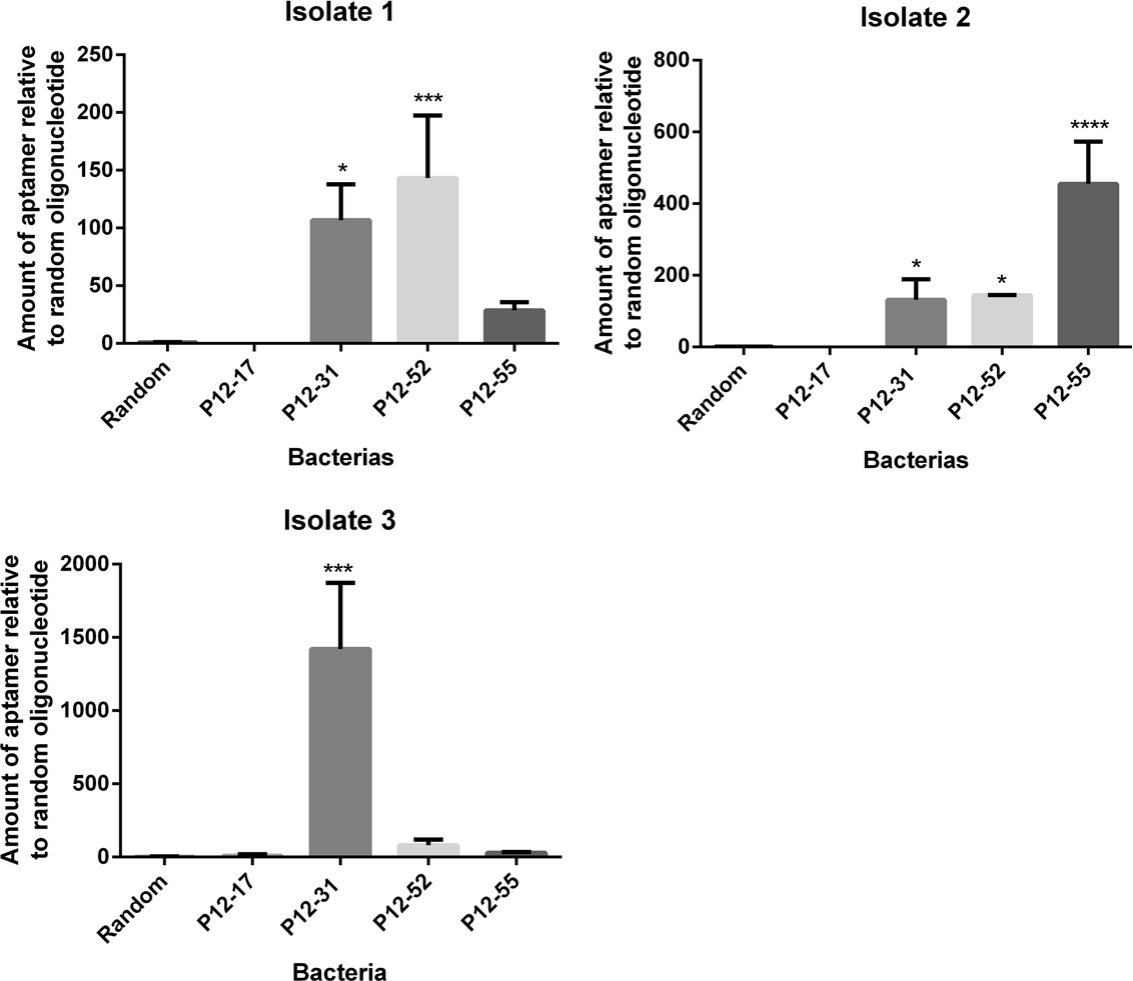
Aptamer binding to meningitis/sepsis-associated *E*. *coli* (MNEC). Aptamers P12-17, P12-31, P12-52 and P12-55 were incubated with live cells from MNEC clinical isolates and then bound aptamers were quantified by qPCR. Data represent mean ± SD of three independent experiments. * p<0.05, *** p<0.001, **** p< 0.0001.

*E*. *coli* serotypes have different cell wall composition, which includes variation in cell wall receptors vary within different *E*. *coli* strains [1]. To assess whether the selected aptamers could recognize *E*. *coli* strains other than ATCC 25922 we evaluated the binding of aptamers P12-17, P12-31, P12-52 and P12-55 to three meningitis/sepsis associated *E*. *coli* (MNEC) clinical isolates. Aptamers P12-31, P12-52 and P12-55 showed higher binding to *E*. *coli* isolates relative to the random oligonucleotide, indicating that the aptamers are able to recognize pathogenic *E*. *coli* strains (Fig 7). On the other hand, P12-17 did not recognize different *E*. *coli* isolates. The aptamers had different binding strength to the distinct bacterial isolates. P12-31 shows stronger binding to isolates 1, 2 and 3 than to *E*. *coli* ATCC 25922. P12-52 show similar binding to isolates 1, 2 and *E*. *coli* ATCC 25922, while it didn’t bind to isolate 3. Finally, P12-55 showed very strong binding to isolate 2, strong binding to *E*. *coli* ATCC 25922 and no significant binding to isolates 1 and 3.

## Discussion

*E*. *coli* strains generate numerous disease conditions with high social and economic impact. Nevertheless, *E*. *coli* detection in the clinic remains suboptimal, with urgent need for faster and more sensitive detection methods, which could be aided by the use of aptamers as diagnostic tools in biosensors devices. Here we used a whole cell-SELEX procedure to select ssDNA aptamers against live *E*. *coli* cells from strain ATCC 25922, and we isolate four aptamers - P12-17, P12-31, P12-52 and P12-55—that bind to these cells with high affinity (Fig 2, Table 2).

Fluorescence analysis suggested that the target molecules recognized by the aptamers described here localize to the bacterial surface. This localization pattern is interesting, and indicates that the aptamers could be used for *E*. *coli* recognition purposes. Also, surface localization is likely to facilitate bacterial enrichment for diagnostics. For numerous aptamers selected for cancer cells or conditioned media (from cancer cell culture), the molecular target recognized by the aptamer was identified, and represented a novel cancer biomarker [69–72]. Aptamers allowed the identification of the alkaline phosphatase placental-like 2 (ALPPL-2) as enriched in pancreatic cancer cells, and a biomarker of pancreatic cancer cell secretomes [69, 71]. Similarly, the use of aptamers showed that the protein tyrosine kinase 7 (PTK7) is enriched in T-cell acute lymphoblastic leukemia [72], and the immunoglobin heavy chain mu in Burkitt’s lymphoma [70]. In the case of *E coli*, aptamers such as those described here could aid in the identification of bacterial serotypes [1].

The aptamer P12-31 has at least ten-fold stronger binding to *E*. *coli* than to the other twelve bacterial species tested here (Fig 5), indicating that this aptamer is highly specific for *E*. *coli*. On the other hand, the selected aptamers P12-17, P12-52 and P12-55 show strong binding to different bacterial species, possibly due to the lack of a negative selection step to exclude aptamers that bind to Gram-negative or Gram-positive bacteria other than *E*. *coli* [65, 73]. Most specificity test for aptamers isolated against *E*. *coli* cells uses bacterial samples with as many as four bacterial species [46, 47, 64, 74]. It remains possible that cross reactivity accounts for part of the binding activity observed here towards non-*E*. *coli* species. Indeed, Bruno and co-workers (2010) reported cross reaction with *S*. *enterica* cells of aptamers selected for *E*. *coli* outer membrane proteins (OMPs) [74].

The fact that the four aptamers recognize different subsets of bacterial species (Fig 5), suggests that the aptamers P12-17, P12-31, P12-52 and P12-55 bind to different molecular markers. Interestingly P12-17 and P12-52 can also recognize Gram-positive bacteria (Fig 5). Gram-positive cells lack the outer lipopolysaccharide membrane that defines bacterial species as Gram-negative; thus, it is possible that P12-17 and P12-52 bind to common groups in different membrane receptors.

Besides being highly specific to *E*. *coli*, P12-31 recognizes other *E*. *coli* subtypes, such as MNEC clinical isolates (Fig 7). Thus, P12-31 is likely to recognize a molecule specific and probably common to the *E*. *coli* strains tested here, and may recognize other *E*. *coli* strains. Differences in the strength of aptamer binding to the *E*. *coli* isolates used here could be explained by differences in the level of expression of target molecules on the bacterial cell surface. In agreement with our results, the aptamer EcA5-27 –selected against the uropathogenic *E*. *coli* NSM59—shows between 8.5 and 56 fold higher affinity to seven uropathogenic *E*. *coli* clinical isolates than to *E*. *coli* laboratory strains such as DH5α, SM10 and BW20338 [22]. In addition, the aptamers selected against fecal *E*. *coli* isolate KCTC 2571 were capable of binding with different affinities to other fecal *E*. *coli* strains, such KCTC 1681, KCTC 2617 and KCTC 2618. [23].

Aptamers against *E*. *coli* have been isolated previously by SELEX against extracellular proteins, OMPs of *E*. *coli* ATCC 8739 or the K88 fimbriae protein of the enterotoxigenic (ETEC) K88, [46, 74], or by cell SELEX against other *E*. *coli* strains such as the laboratory strain DH5α, enterohemorrhagic O157:H7, uropathogenic NSM59 and fecal *E*. *coli* isolate KCTC 2571 [22, 23, 75–77]. However, to our knowledge, this is the first study to report the binding of aptamers to MNEC. Importantly, our results show that some of the aptamers described here recognize pathogenic strains with affinity and specificity compatible with their potential use in clinical diagnosis and therapheutic applications.

## Supporting Information

S1 Fig Native polyacrylamide gel electrophoresis (6%) showing PCR products that resulted from the use of the 88-bp nucleotide starting library as a template. PCR results of conventional PCR. Neg negative control from conventional PCR experiment. aPCR, results from asymmetric PCR (aPCR) performed in the conditions used during SELEX. The position of double stranded (88 bp) or singles stranded (88nt) product is indicated on the left.
(PDF)

S2 Fig Binding of P12-31 to live whole cells from different bacterial species relative to that of the random oligonucleotide used as a control. The differences between P12-31 binding to *E coli* and to all other bacterial species were statistically significant (** p<0.005).
(PDF)

S3 Fig Stable conformers of the P12-17 mfold prediction [63]. Conserved stem loops are highlighted in bold.
(PDF)

S4 Fig Stable conformers of P12-52 predicted by the mfold algoritm [63]. Conserved stem loops are highlighted in bold.
(PDF)

S1 Table Conditions used in iteractive rounds of *E*. *coli* SELEX. *Correspond to the library ssDNA population. ** Washes were done with selection buffer (PBS containing 1.4 mM MgCl_2_).
(PDF)

S2 Table Statistical P-values (t-test) for the differences in binding of aptamer P12-31 to *E*. *coli* vs. different bacterial species. (PDF)
