# Supplementary figures and images for: Isolation of an Aptamer that Binds Specifically to E. coli
Source: PLoS One. 2016 Apr 22;11(4):e0153637. doi: 10.1371/journal.pone.0153637 (PMC4841571; doi:10.1371/journal.pone.0153637)

88 bp —  
88 nt —

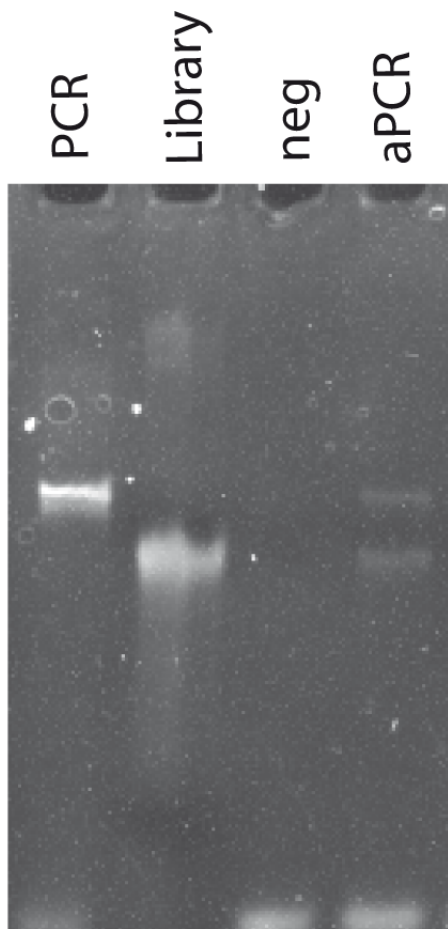

Supplement: S1 Fig — PCR results of conventional PCR. Neg negative control from conventional PCR experiment. aPCR, results from asymmetric PCR (aPCR) performed in the conditions used during SELEX. The position of double stranded (88 bp) or singles stranded (88nt) product is indicated on the left. (PDF) [file pone.0153637.s001.pdf]

## P12-31

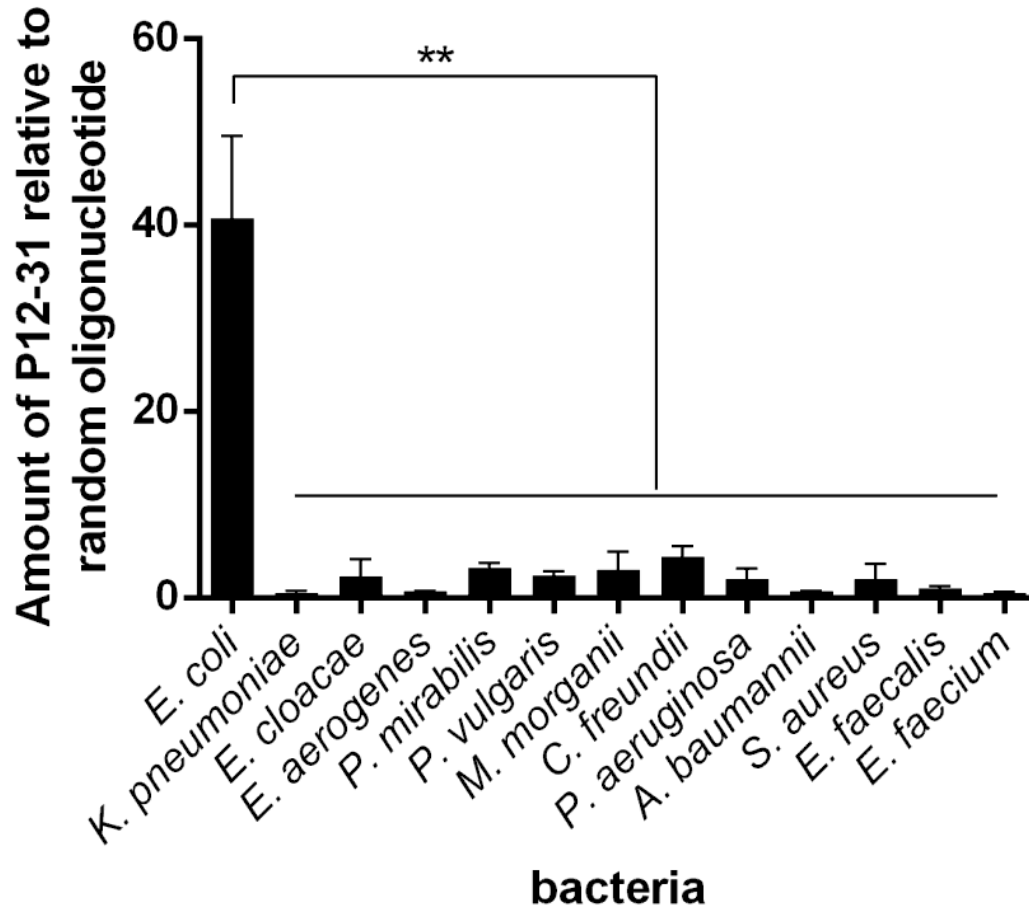

Supplement: S2 Fig — The differences between P12-31 binding to E coli and to all other bacterial species were statistically significant (** p<0.005). (PDF) [file pone.0153637.s002.pdf]

P12-17

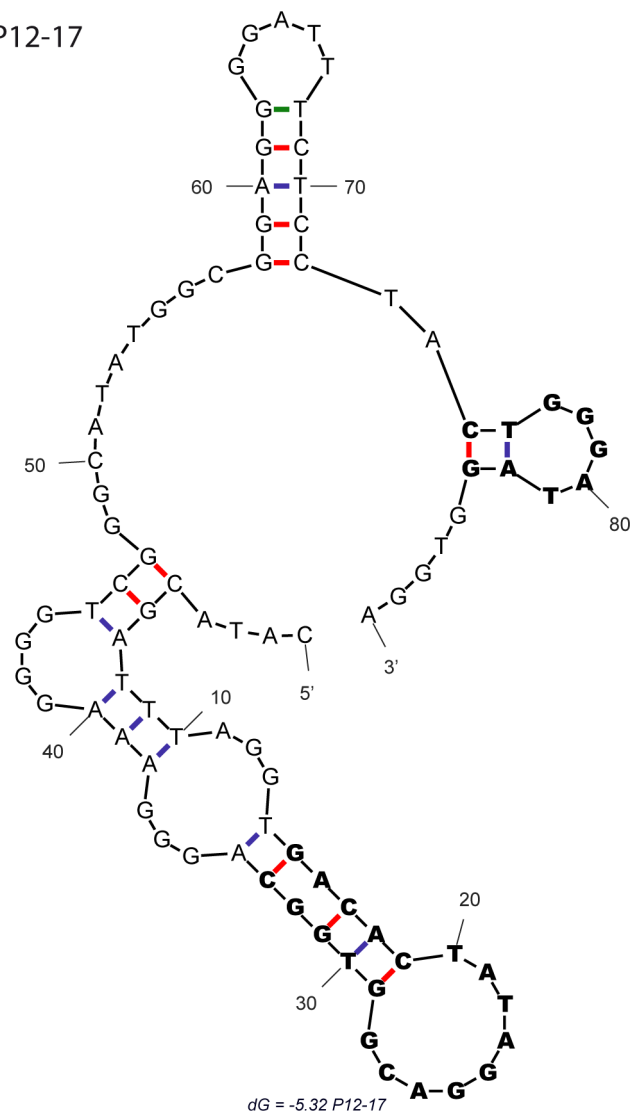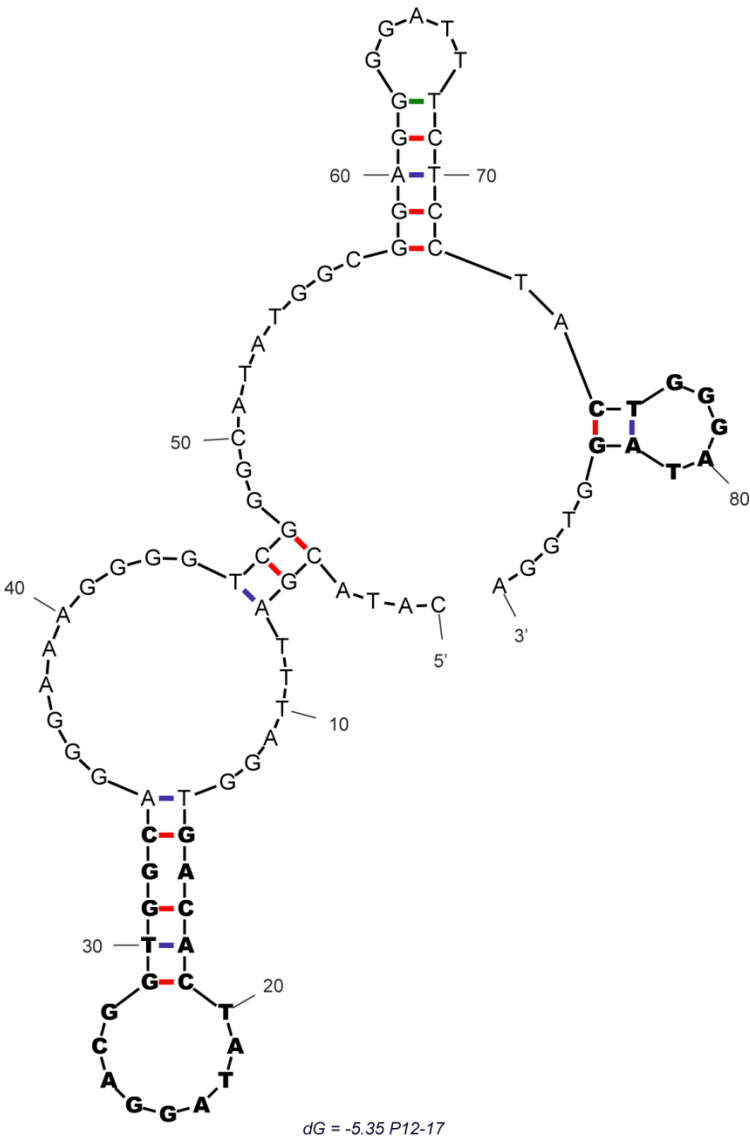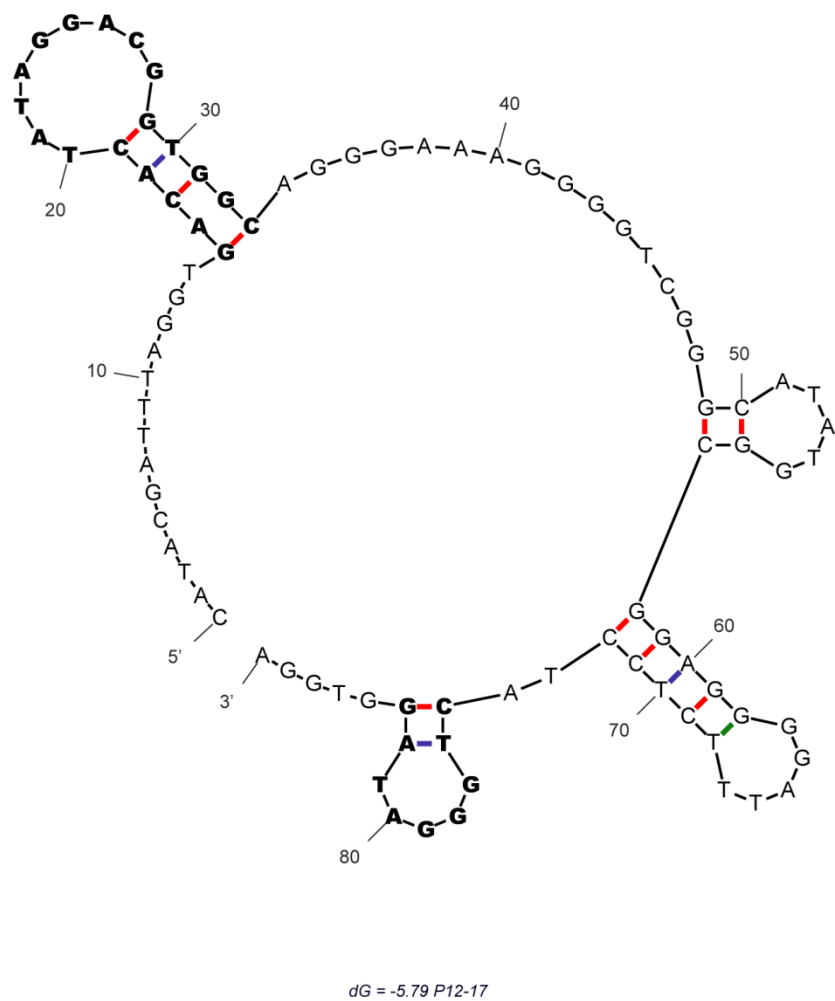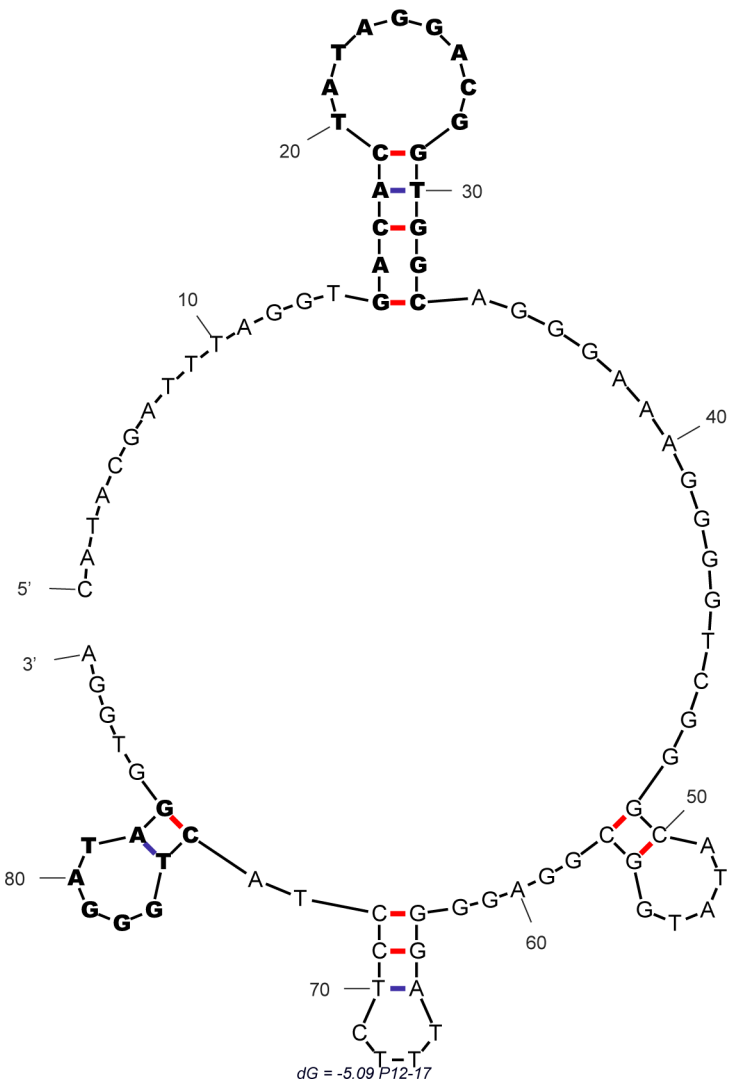

Supplement: S3 Fig — Conserved stem loops are highlighted in bold. (PDF) [file pone.0153637.s003.pdf]

P12-52

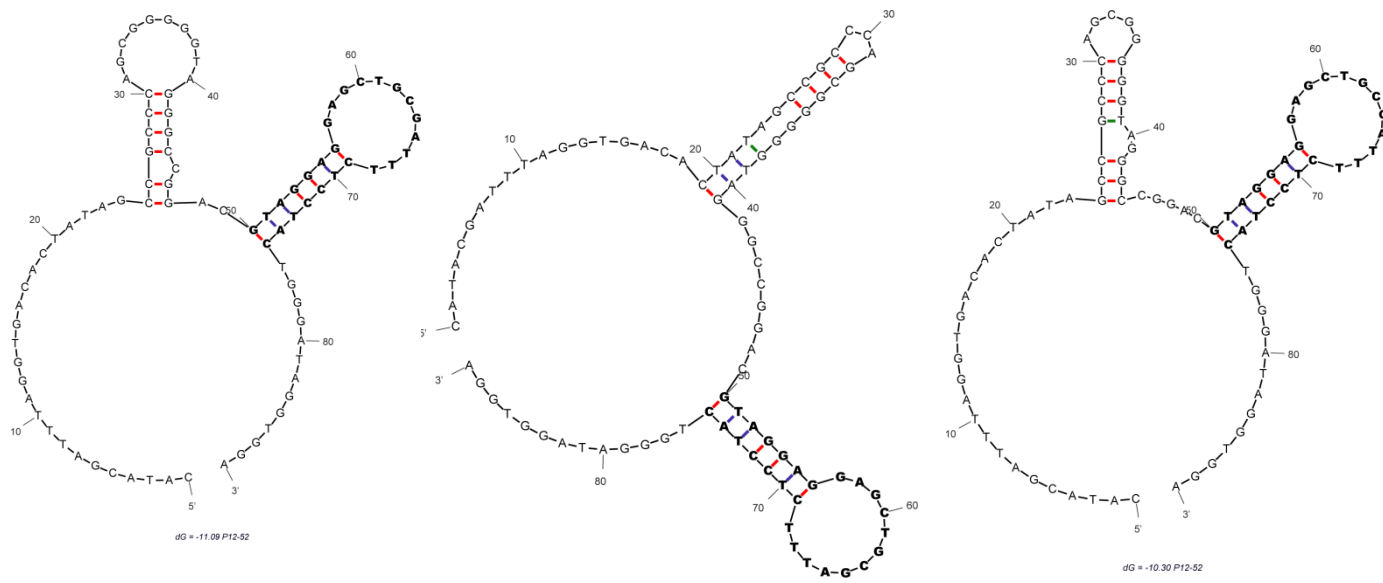

dG = -11.00 P12-52

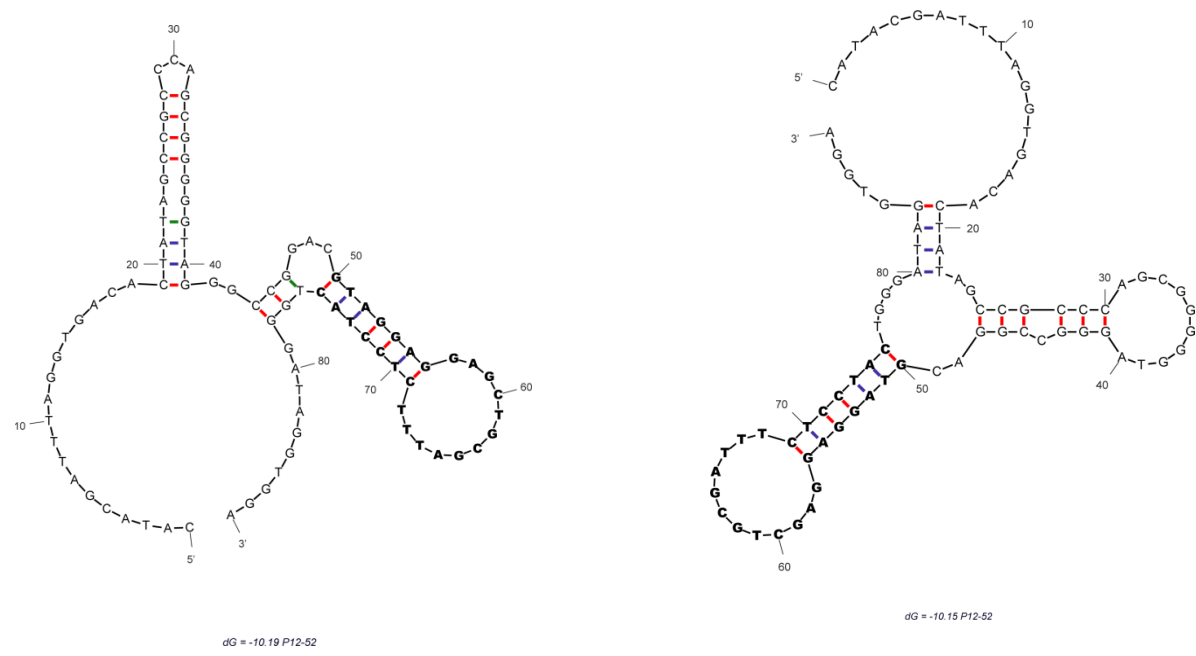

Supplement: S4 Fig — Conserved stem loops are highlighted in bold. (PDF) [file pone.0153637.s004.pdf]
