# Supplementary material for: Isolation of an Aptamer that Binds Specifically to E. coli
Source: PLoS One. 2016 Apr 22;11(4):e0153637. doi: 10.1371/journal.pone.0153637 (PMC4841571; doi:10.1371/journal.pone.0153637)
Supplement: S1 Table — *Correspond to the library ssDNA population. ** Washes were done with selection buffer (PBS containing 1.4 mM MgCl2). (PDF) [file pone.0153637.s005.pdf]

TABLE S1: Conditions used in iterative rounds of *E. coli* SELEX.

Selection Round	Bacteria (cels/mL)	Aptamer (pmols)	Competitor	Binding time (min)	Washes (time, number, volume) **
1	10^7	1500 (4.59 x 10^{14} molecules) *	____	45	3 min, 1 x, 1 mL
2	10^7	6.8	____	45	3 min, 1 x, 1 mL
3	10^7	34.4	0.1 µg/µl salmon sperm DNA	45	3 min, 1 x, 1 mL
4	10^6	75.5	0.1 µg/µl salmon sperm DNA	45	3 min, 1 x, 1 mL
5	10^6	22	0.1 µg/µl salmon sperm DNA, 0.05 µg/µl BSA	45	3 min, 3 x, 1 mL
6	10^5	12	0.1 µg/µl salmon sperm DNA, BSA 0.05 µg/µl	45	3 min, 3 x, 1 mL
7	10^4	17	0.05 µg/µl BSA	45	3 min, 3 x, 1 mL
8	10^4	6.8	0.2 µg/µl salmon sperm DNA, 0.1 µg/µl BSA	45	3 min, 3 x, 1 mL
9	10^4	6.1	0.2 µg/µl salmon sperm DNA, 0.1 µg/µl BSA	45	20 min,3 x, 1 mL
10	10^3	8.9	0.2 µg/µl salmon sperm DNA, 0.1 µg/µl BSA	30	20 min,2 x, 1 mL
11	10^3	6.5	0.2 µg/µl salmon sperm DNA	30	20 min, 6x, 1 mL
12	10^3	4.4	0.2 µg/µl BSA	30	20 min, 6 x, 1 mL
		4.2			

*Correspond to the library ssDNA population.

** Washes were done with selection buffer (PBS containing 1.4 mM $MgCl_2$).
