# Supplementary material for: Isolation of an Aptamer that Binds Specifically to E. coli
Source: PLoS One. 2016 Apr 22;11(4):e0153637. doi: 10.1371/journal.pone.0153637 (PMC4841571; doi:10.1371/journal.pone.0153637)
Supplement: S2 Table — (PDF) [file pone.0153637.s006.pdf]

TABLE S2: Statistical P-values (t-test) for the differences in binding of aptamer P12-31 to *E. coli* vs. different bacterial species.

Bacteria	P-value
K. Pneumoniae	0.0014
E. cloacae	0.0018
E. aerogenes	0.0014
P. mirabilis	0.0018
P. vulgaris	0.0017
M. morganii	0.0019
C. freundii	0.0021
P. aeruginosa	0.0017
A. baumannii	0.0014
S. aureus	0.0017
E. faecalis	0.0015
E. faecium	0.0014
